# Prenatal Lead Exposure and Schizophrenia: Further Evidence and More Neurobiological Connections

**DOI:** 10.1289/ehp.0800484

**Published:** 2009-05

**Authors:** Tomás R. Guilarte

**Affiliations:** Environmental Health Sciences, Johns Hopkins Bloomberg School of Public Health, Baltimore, Maryland, E-mail: tguilart@jhsph.edu

In 2004, Opler et al. published a study in *Environmental Health Perspectives* (*EHP*) suggesting an association between prenatal lead (Pb^2+^) exposure and schizophrenia (Opler et al. 2004). In the November 2008 issue of *EHP*, Opler et al. (2008) further supported this association using a different cohort of subjects. In a letter published in *EHP* in 2004 (Guilarte 2004), I indicated that a plausible neurobiological connection between prenatal Pb^2+^ exposure and schizophrenia may be that Pb^2+^ is a potent antagonist of the *N*-methyl d-aspartate (NMDA) receptor (NMDAR), and NMDAR hypofunction is thought to be involved in the pathophysiology of the disease. Since then, another plausible neurobiological connection has surfaced, and this relates to hippo campal neurogenesis. Neurogenesis occurs not only during development but is also prominent in the adult brain (Laplagne et al. 2006). A well-characterized neurogenic zone in the adult brain is the subgranular zone of the dentate gyrus (DG) in the hippocampus (Zhao et al. 2008). Although the significance of newly born neurons in the adult hippocampus is currently under investigation, the overwhelming evidence supports a role in hippocampus-dependent learning (Dupret et al. 2008; Imayoshi et al. 2008).

Schizophrenia patients express cognitive deficits that may be related to hippocampal dysfunction (Gothelf et al. 2000; Sweatt 2004). So, what is the new neurobiological connection between Pb^2+^ exposure and schizophrenia? Recent evidence indicates that neurogenesis is decreased in schizophrenia patients, and this decrease may contribute to their cognitive dysfunction (Kempermann et al. 2008; Reif et al. 2006). In an animal model using the NMDAR antagonist phencyclidine (PCP) to induce schizophrenia-like symptoms in mice, Maeda et al. (2007) observed reduced DG neurogenesis that was reversed by the atypical anti-psychotic drug clozapine. Co-administration of d-serine and glycine also inhibited the PCP-induced decrease in neurogenesis. PCP, like Pb^2+^, is an NMDAR antagonist, and D-serine and glycine activate NMDAR; this suggests that chronic NMDAR hypofunction decreases neurogenesis in the hippo campus, an observation consistent with my comments in 2004 (Guilarte 2004). Models of developmental Pb^2+^ exposure have also shown decreased DG neurogenesis and are associated with deficits in learning (Jaako-Movits et al. 2005; Verina et al. 2007). Therefore, reduced DG neuro genesis appears to be a common factor in schizophrenia and in animal models of schizophrenia and developmental Pb^2+^ exposure.

Schizophrenia is a neurodevelopmental disorder that is expressed later in life. Pb^2+^ is a neurotoxicant that is known to cause developmental abnormalities. Animal models of developmental Pb^2+^ exposure express a behavioral phenotype with features that overlap with those in animal models of schizophrenia, including increased spontaneous activity, decreased social interaction, and learning deficits (Moreira et al. 2001; Nihei et al. 2000). Also, some of the behavioral effects described in adolescents with early-life Pb^2+^ exposure are similar to those expressed in schizophrenia patients (Opler and Susser 2005). Thus, although the environmental causes of schizophrenia have not evaluated environmental toxicants, the emerging evidence from the human studies by Opler and colleagues and animal studies suggest that prenatal Pb^2+^ exposure may be an environmental risk factor for schizophrenia.

## Editor’s note

In accordance with journal policy, Opler et al. were asked whether they wanted to respond to this letter, but they chose not to do so.
